# High nuclear level of Vav1 is a positive prognostic factor in early invasive breast tumors: a role in modulating genes related to the efficiency of metastatic process

**DOI:** 10.18632/oncotarget.2011

**Published:** 2014-05-25

**Authors:** Silvia Grassilli, Federica Brugnoli, Rossano Lattanzio, Cosmo Rossi, Letizia Perracchio, Marcella Mottolese, Marco Marchisio, Maria Palomba, Ervin Nika, Pier Giorgio Natali, Mauro Piantelli, Silvano Capitani, Valeria Bertagnolo

**Affiliations:** ^1^ Section of Anatomy and Histology, Department of Morphology, Surgery and Experimental Medicine, University of Ferrara, Ferrara, Italy; ^2^ Department of Experimental and Clinical Sciences, University “G. d'Annunzio” Chieti, Italy; ^3^ Center of Excellence for Research on Aging, Foundation University “G. d'Annunzio”, Chieti, Italy; ^4^ Department of Biomorphology, University “G. D'Annunzio” Chieti, Italy; ^5^ “Regina Elena” Cancer Institute, Rome, Italy; ^6^ CINBO Laboratories, University “G. D'Annunzio”, Chieti, Italy; ^7^ LTTA Centre, University of Ferrara, Ferrara, Italy

**Keywords:** breast cancer, Vav1, cell nucleus, metastasis

## Abstract

Vav1 is one of the signalling proteins normally restricted to hematopoietic cells that results ectopically expressed in solid tumors, including breast cancer. By immunohistochemical analysis on TMAs containing invasive breast tumor from patients without lymph node involvement, we have found that Vav1 is expressed in almost all investigated cancers and shows a peculiar localization inside the nucleus of tumor cells. High amounts of nuclear Vav1 are positively correlated with low incidence of relapse, regardless phenotype and molecular subtype of breast neoplasia. In particular, Kaplan-Meier plots showed an elevated risk of distant metastasis in patients with low Vav1 expression compared with patients with high Vav1 expression in their tumors. Experiments performed with breast tumor-derived cells indicated that Vav1 negatively modulates their invasiveness *in vitro* and their metastatic efficiency *in vivo,* possibly by affecting the expression of genes involved in invasion and/or metastasis of breast tumors. Since the high heterogeneity of breast tumors makes difficult to predict the evolution of early breast neoplasias, the evaluation of nuclear Vav1 levels may help in the characterization and management of early breast cancer patients. In particular, Vav1 may serve as a prognostic biomarker and a target for new therapies aimed to prevent breast cancer progression.

## INTRODUCTION

The chances of disease-free survival of breast cancer patients increased in the last few decades only for breast cancers diagnosed and treated at an early stage, while for breast cancer metastasized to other organs, the therapeutic options are still very limited [1]. A number of deregulated signalling molecules are variously correlated with the malignant progression of breast tumors, but the high intrinsic heterogeneity that characterizes this neoplasia makes difficult to establish unique correlations and to clearly identify molecules useful to predict, and hopefully to prevent, breast cancer recurrence [1, 2].

**Table 1:** Patients and tumor characteristics (*n* = 137)

Variable	Value (%)
Age at diagnosis (yr)	
Median	55.6
<50	50 (36.5)
50-65	52 (38.0)
>65	35 (25.5)
Menopausal status	
Pre/perimenopausal	51 (37.2)
Postmenopausal	86 (62.8)
Tumor size	
≤ 2 cm	97 (70.8)
> 2 cm	40 (29.2)
Histotypes	
Lobular carcinoma	106 (77.4)
Ductal carcinoma	20 (14.6)
Other	11 (8.0)
Tumor grade	
1	18 (13.1)
2	74 (54.0)
3	45 (32.9)
ER	
Negative	33 (24.1)
Positive	104 (75.9)
PR	
Negative	43 (31.4)
Positive	94 (68.6)
Ki-67	
Low	76 (55.5)
High	61 (44.5)
HER-2	
Positive	121 (88.3)
Negative	16 (11.7)
Patient outcome	
Without recurrence	113 (82.5)
Local recurrence	8 (5.8)
Distant recurrence	16 (11.7)

Vav1 is the sole of the 3 members of the Vav family to be physiologically expressed exclusively in haematopoietic cells in which, in parallel with the best known function as a guanosine exchange factor (GEF) mainly devoted to the rearrangement of actin cytoskeleton [3], it acts as an adaptor/regulator molecule in both cytoplasm and nuclear compartments [4-6]. Vav1 plays a crucial role in maturation and function of both myeloid and lymphoid cells [6] and its expression in non-hematopoietic tissues, including breast, is associated with a tumoral phenotype [7-12]. Contrarily to other solid tumors, in which Vav1 positively correlates with malignancy [8, 12], the *Vav1* transcript in breast cancers seems to be higher in tumors from patients that remained disease free than in patients who developed recurrence [13]. On the other hand, no correlations between the expression levels of Vav1 protein in primary tissues and the clinicopathological features of breast tumor have been reported.

The contribution of Vav1 in tumorigenic properties of solid tumors has only been assigned so far to its GEF activity [8, 10, 11] and, in breast tumor-derived cell lines, a dual potential role of Vav1 as an oncogenic stress activator as well as an apoptotic inducer dependent from p53 availability was reported [14]. Even though in hematopoietic cells Vav1 shows also a nuclear localization and plays a peculiar GEF-independent role inside the nuclear compartment of tumoral promyelocytes [6], in breast cancer as in other solid neoplasia, roles of Vav1 connected to its intracellular localization have not been described.

This work was aimed to establish the significance of Vav1 expression in breast tumor cells, in terms of tumor progression measured as risk of recurrence in patients with T1-T2, N0, M0 breast cancer. The results indicate that, in early breast cancers patients, high nuclear expression of Vav1 in tumor cells is an independent prognostic factor associated with low risk of distant metastases. Experiments performed with breast tumor-derived cell lines and *in vivo* models indicate that Vav1 may reduce the malignant potential of breast tumor cells possibly by affecting the expression of genes involved in breast tumor progression.

## RESULTS

### Nuclear Vav1 in breast tumor tissues

**Figure 1:**
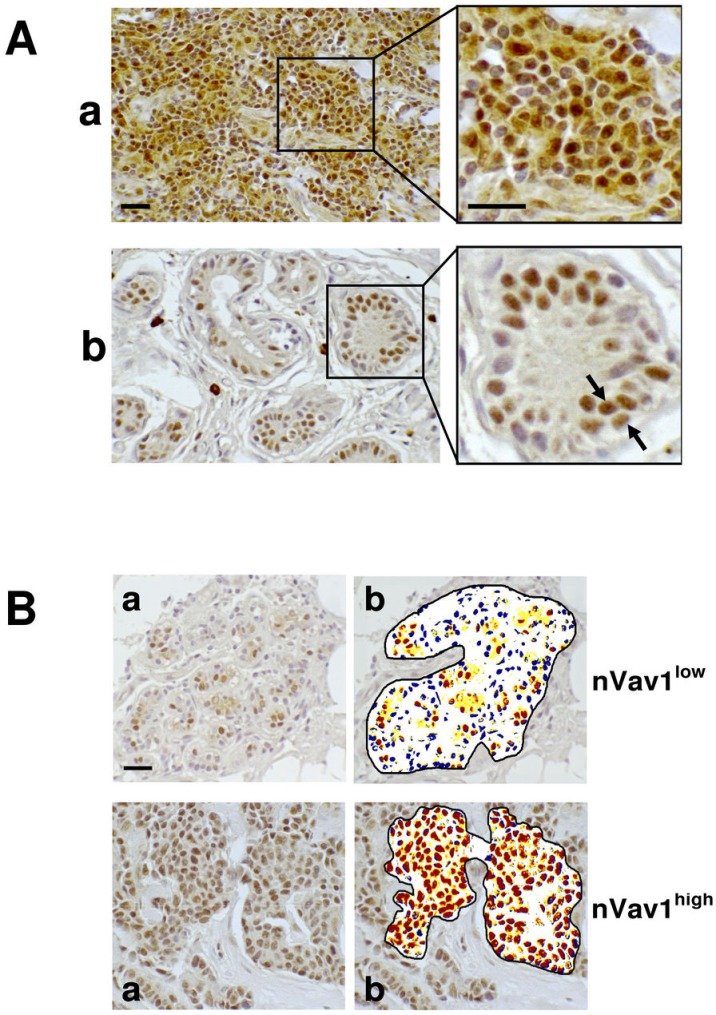
Nuclear expression of Vav1 in breast cancer tissues (A) Deparaffinized TMA sections of invasive breast tumors were incubated with a polyclonal anti-Vav1 antibody and observed with an optical microscope after chromogen reaction. a: staining of cytoplasm and nucleus; b: exclusive staining of nuclei. The arrows indicate a dotted nuclear staining. (B) Representative images of analysis performed with ImageScope software (b) on deparaffinized breast cancer sections showing low (yellow) and high (red) levels of nuclear staining with the anti-Vav1 antibody (a). In blue the unstained nuclei are shown. nVav1^low^: representative tissue sample showing less than 60% of nuclei expressing high levels of Vav1; nVav1^high^: representative tissue sample showing more than 60% of nuclei expressing high levels of Vav1. Bar = 20 μm.

Immunohistochemical analysis with the anti-Vav1 antibody performed on TMAs revealed that almost all tumor tissues express Vav1, since the protein was absent in only 5 out of 137 examined early breast tumors. Immunoreactivity for Vav1 was found primarily in the nucleus of tumor cells, with or without concomitant cytoplasmic staining (Fig. 1A). The number of Vav1-positive nuclei ranged from 5% to 98%, with a mean ± SE of 65.8 ± 7.9. The staining of positive nuclei was not homogeneous and, in some cases, a distinct dotted picture was observed (Figure 1A). The levels of nuclear staining for Vav1 were quantified as reported in the “Materials and Methods” section and, based on the number of highly positive nuclei, 51% of tumors were classified as highly expressing (nVav1^high^) and the remaining tumors as low expressing (nVav1^low^) (Figure 1B).

**Figure 2:**
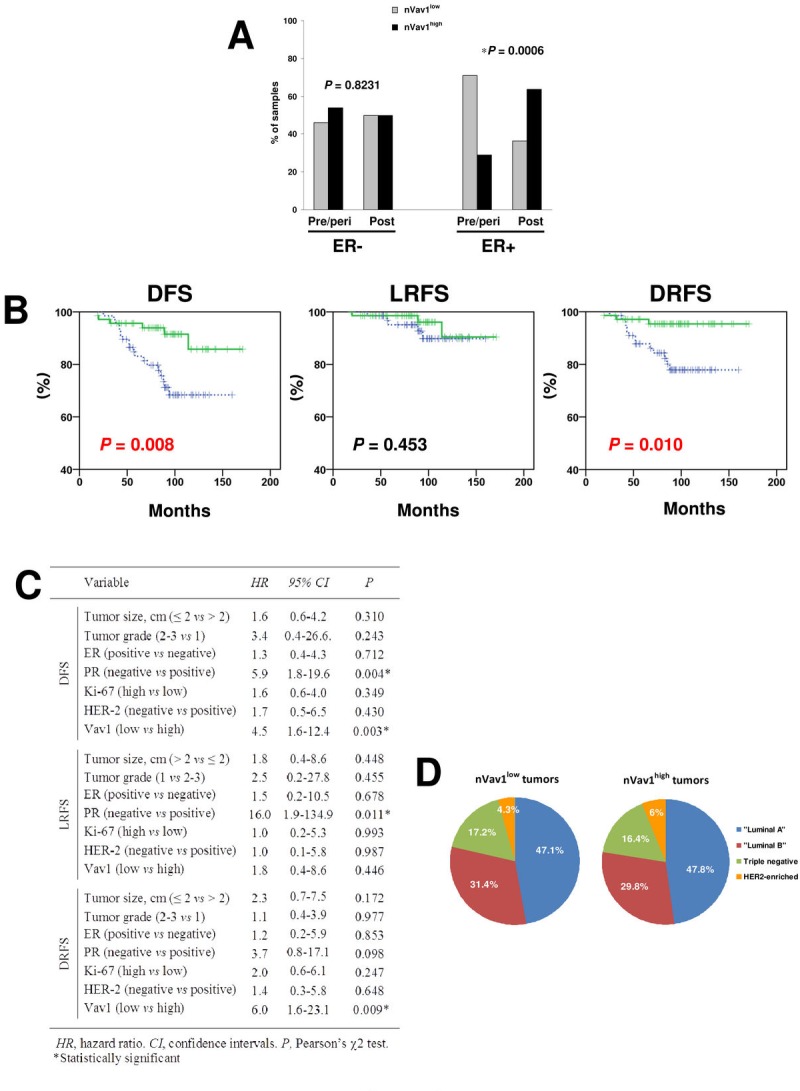
Relationship between levels of nuclear Vav1 and clinicopathological features of breast tumors (A) Percentages of tumors from pre/perimenopausal (pre/peri) and post-menopausal (post) patients expressing low (nVav1^low^) or high (nVav1^high^) levels of nuclear Vav1. A separate analysis was performed in ER positive (ER+) and ER negative (ER-) breast cancer samples. The asterisk indicates statistically significant differences (P<0.05). (B) Kaplan-Meier curves showing disease-free survival (DFS), local relapse-free survival (LRFS) and distant relapse-free survival (DRFS) of early breast tumor patients according to high (green solid line) and low (blue dashed line) nuclear expression of Vav1 (P<0.05). (C) Multivariate analysis of nuclear expression of Vav1. (D) Distribution of tumors expressing respectively low (nVav1^low^) and high (nVav1^high)^ levels of nuclear Vav1 in the different clinicopathological subtypes of breast cancer.

The relationships among the amounts of Vav1 inside the nuclear compartment of tumor cells and tumor size, histotype, tumor grade, proliferation index and receptors status were not statistically significant (Table 2). On the contrary, a significant correlation was observed between the nuclear levels of Vav1 and the age at diagnosis (*P* = 0.006), since the majority of patients <50 years old showed primary tumors with low levels of nuclear Vav1 and the tumors from 50-65 years patients were mostly nVav1^high^. On the other hand, patients >65 years showed primary tumors equally expressing low and high levels of nuclear Vav1 (Table 2). A significant correlation was shown between the amounts of Vav1 at nuclear level and menopausal status (*P* = 0.004), reflecting the age since the majority of pre/perimenopausal patients showed primary tumors with low nuclear expression of Vav1 and, on the contrary, tumors from postmenopausal patients showed high levels of the protein at nuclear level (Table 2). This correlation is strongly dependent on the ER status of primary tumors. In fact, in ER- tumors, no difference in Vav1 amounts at nuclear level was observed between tumors from patients at pre/post menopausal status while, in ER+ tumors, a strong correlation between high nuclear amount of the protein and menopausal status was observed (Figure 2A). The same results (for ER- tumors, *P* = 0.885 and for ER+ tumors *P* = 0.001) were obtained when the positivity for ER was re-evaluated according to the current trend to consider as ER-positive tumors expressing nuclear staining in at least 1% of cells [15].

**Table 2:** Nuclear levels of Vav1 according to clinico-pathological parameters in T1-T2, N0 breast cancer patients (*n* = 137)

Variable	nVav1^low^ (n %)	nVav1^high^ (n %)	P	P°
Age at diagnosis (yr)				
<50	33 (66.0)	17 (34.0)		0.006*
50-65	18 (34.6)	34 (65.4)		
>65	16 (45.7)	19 (54.3)		
Menopausal status				
Pre/perimenopausal	33 (64.7)	18 (35.3)	0.004*	
Postmenopausal	34 (39.5)	52 (60.5)		
Tumor size				
≤ 2 cm	49 (50.5)	48 (49.5)	0.597	
> 2 cm	18 (45.0)	22 (55.0)		
Histotypes				
Ductal carcinoma	50 (47.2)	56 (52.8)		0.596
Lobular carcinoma	10 (50.0)	10 (50.0)		
Other	7 (63.6)	4 (36.4)		
Tumor grade				
1	10 (55.5)	8 (44.5)		0.720
2	34 (45.9)	40 (50.1)		
3	23 (51.1)	22 (48.9)		
ER				
Negative	16 (48.5)	17 (51.5)	1.000	
Positive	51 (49.0)	53 (51.0)		
PR				
Negative	20 (47.4)	23 (53.6)	0.708	
Positive	47 (50.0)	47 (50.0)		
Ki-67				
Low	36 (47.4)	40 (52.6)	0.689	
High	31 (50.8)	30 (49.2)		
HER-2				
Negative	58 (47.9)	63 (52.1)	0.532	
Positive	9 (56.2)	7 (43.8)		
Patient outcome				
Without recurrence	49 (43.4)	64 (56.6)		0.013*
Local recurrence	5 (62.5)	3 (37.5)		
Distant recurrence	13 (81.2)	3 (18.8)		

*P*: Pearson 's χ^2^ test.

*P°:* Fisher exact probability test.

* Statistically significant.

The analysis of the relationship between nVav1 and tumor recurrence resulted significant (*P* = 0.013), since 13 out of 16 patients (81.2%) who developed distant metastasis showed primary tumors with nVav1^low^ (Table 2). Kaplan Meier analyses illustrated a significant (*P* = 0.008) association of high nuclear expressions of Vav1 with higher disease free survival (DFS) rate (Figure 2B). In particular, nVav1^high^ tumors were associated with a significantly lower incidence of distant relapse (*P* = 0.010) while no significant association with local recurrence was found (Figure 2B).

Multivariate analyses adjusted for other prognostic factors revealed that low nuclear expression of Vav1 and absence of PR were prognostic factor influencing DFS (HR = 4.5; 95% CI, 1.6-12.4; *P* = 0.003 and HR=5.9; 95% CI, 1.8-19.6; *P* = 0.004, respectively). However, low expression of Vav1 was the only independent factor influencing distant relapse free survival (DRFS) (HR = 6.0: 95% CI, 1.6-23.1; *P* = 0.009), but not local relapse free survival (LRFS) (Figure 2C).

On the basis of immunohistochemical definition of positivity for ER, PR, HER-2 and of levels of the Ki-67 labeling index, tumors were classified into clinicopathological subtypes following the criteria described by Goldhirsch et al. [16]. This allowed to identify 65 “Luminal A” (47.4%), 42 “Luminal B” (30.7%), 7 “HER2 enriched” (5.1%), and 23 “Triple negative” (16.8%) disease subtypes, that do not significantly differ for their nuclear positivity to Vav1 (Figure 2D).

### Expression of Vav1 in breast tumor-derived cell lines

**Figure 3:**
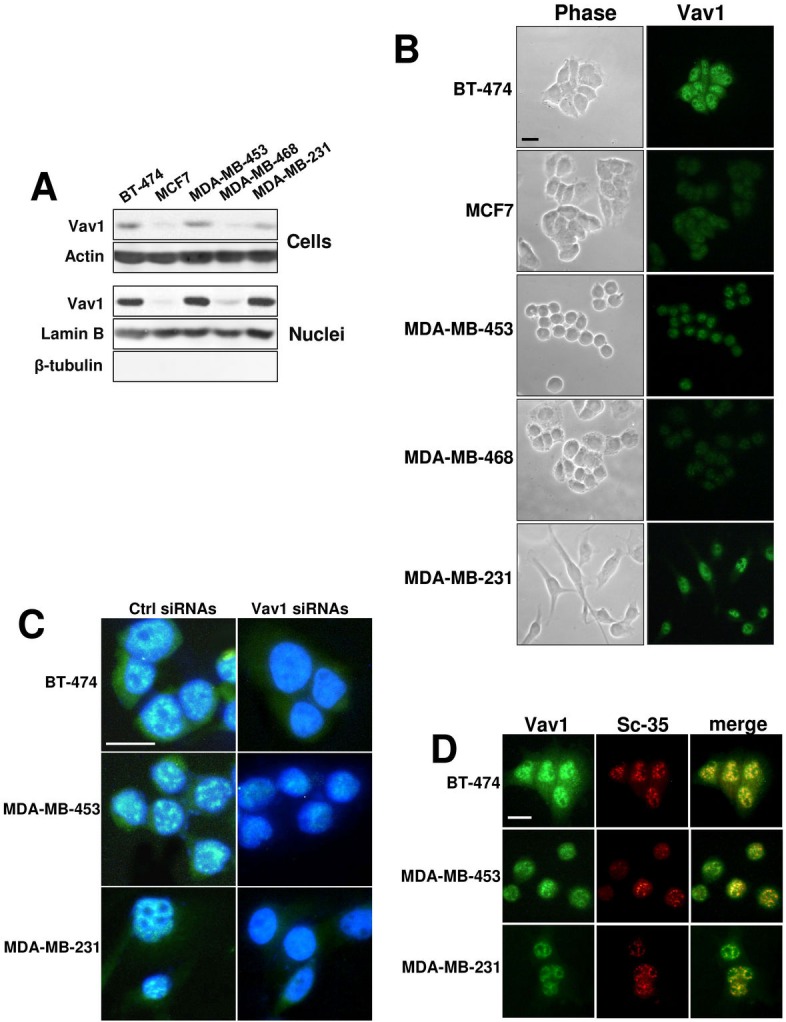
Nuclear Vav1 in breast cancer-derived cell lines (A) Western blot analysis with the anti-Vav1 antibody on total cellular and nuclear lysates from BT-474, MCF7, MDA-MB-453, MDA-MB-468 and MDA-MB-231 breast cancer-derived cells. Cellular and nuclear lysates were also analyzed, respectively, for Actin and Lamin B content, as internal controls of loaded proteins. Nuclear lysates were analyzed for β-tubulin, as a control of the absence of cytoplasm contamination. (B) Representative phase-contrast (Phase) and fluorescence microscopy (Vav1) images of BT-474, MCF7, MDA-MB-453, MDA-MB-468 and MDA-MB-231 cells subjected to immunocytochemical analysis with the anti-Vav1 antibody. (C) Immunocytochemical analysis performed with the anti-Vav1 antibody on BT-474, MDA-MB-453 and MDA-MB-231 cells in which Vav1 expression was forcedly silenced by means of specific siRNAs (Vav1 siRNAs). After staining of the nucleus with 4,6-diamino-2-phenylindole (DAPI), merged anti-Vav1/DAPI images were obtained and analyzed by confocal microscopy. A non-silencing scramble siRNAs was used as a control (ctrl siRNAs). (D) BT-474, MDA-MB-453 and MDA-MB-231 cells were subjected to immunocytochemical analysis with both anti-Vav1 and anti-SC-35 antibodies. Merged images are shown to indicate the co-localization of Vav1 (green) with SC-35 (red) proteins. Bar = 20 μm. All the data are representative of three experiments.

Immunochemical analysis with the anti-Vav1 antibody was performed on 5 breast cancer-derived cell lines (BT-474, MCF7, MDA-MB-453, MDA-MB-468 and MDA-MB-231) with different morphology, immunoprofile and invasive properties, representing the most frequent subtypes of breast tumors. As shown in Figure 3A, Vav1 is expressed in all cell lines, even if MCF7 and MDA-MB-468 cells show a very low amount of the protein. When the immunochemical analysis was performed on highly purified nuclei, apart from MCF7 and MDA-MB-468 cells, a considerable amount of the protein was revealed, indicative of the major localization of Vav1 at nuclear level (Figure 3A).

Immunocytochemical analysis with the same anti-Vav1 antibody was performed on glass slide growing cells. The results, shown in Figure 3B, confirmed the immunochemical data, since Vav1 staining was revealed mainly at nuclear level and with much less intensity in MCF7 and MDA-MB-468 cells. The expression of Vav1 at nuclear level is independent from cell morphology, since of the two analyzed cell lines with an epithelial morphology, BT-474 show high levels of nuclear Vav1 and MCF7 are almost negative. Of the two cell lines with a round morphology, MDA-MB-453 show a Vav1 expression higher than MDA-MB-468. MDA-MB-231 cells, which show a spindle morphology, express nuclear Vav1 at intermediate levels (Figure 3B). Inside the nuclear compartment of all cell lines, Vav1 accumulates as discrete, dot-like substructures and is excluded from the rounded dominions that resembled nucleoli (Figure 3B).

The confocal analysis of cells in which the expression of Vav1 was down-modulated with specific siRNAs (Supplementary Figure 1A) confirmed on one hand the nuclear localization of the protein, mainly in speckled structures in areas of DAPI light staining, and on the other allowed to establish the specificity of the anti-Vav1 antibody employed (Figure 3C).

Co-localization experiments of Vav1 with the speckle marker SC-35 were performed in all the 3 cell lines expressing high levels of Vav1, showing the overlapping between the two proteins, indicative of the localization of Vav1 in specific subnuclear structures (Figure 3D).

### Vav1-related reduction of invasive/metastatic properties

**Figure 4:**
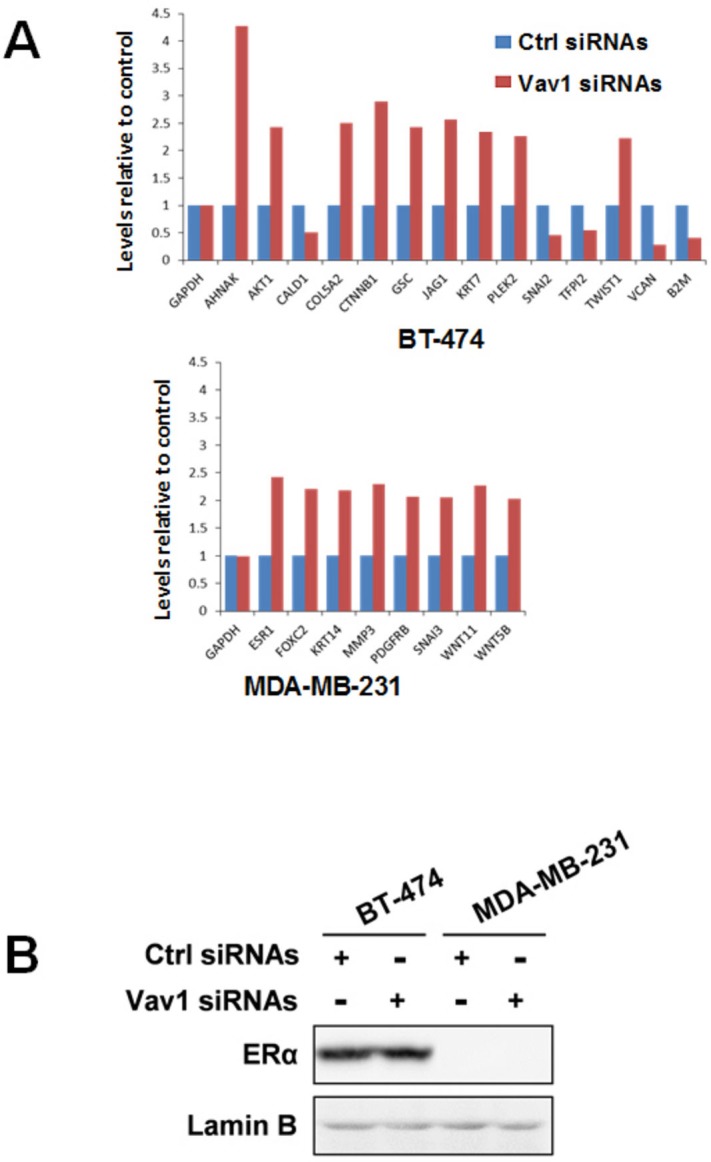
Role of Vav1 in modulating gene expression in breast tumor cells (A) Total RNA isolated from BT-474 and MDA-MB-231 cells transfected with non-silencing RNAs (ctrl siRNAs) or with siRNAs specific for Vav1 (Vav1 siRNAs) was hybridized to a human epithelial to mesenchymal transition (EMT) PCR array. Changes in the gene expression were analyzed by using SABioscience software with GAPDH as a control for normalization. Only genes whose expression was more than two fold differentially expressed are listed. The experiment was performed in triplicate. (B) Cells in the same experimental conditions were subjected to Western Blot analysis with an anti-ERα antibody. Lamin B was used as internal control of loaded proteins. The data are representative of three experiments.

Considering the main nuclear localization of Vav1 in breast tumor cells and its correlation with their ability to give rise to distant metastasis, the possible role of Vav1 in modulating the expression of genes involved in tumor progression was investigated. With this intent, the non-metastatic BT-474 and the moderately metastatic MDA-MB-231 cells, both expressing high levels of Vav1 (Figure 3), were transiently transfected with siRNAs specific for Vav1 (Supplementary Figure 1A) and analyzed for the expression of a focused panel of genes (Supplementary Table 1) variously involved in the different steps of the metastatic process of solid tumors. Of the 84 analyzed genes, 9 were up-regulated and 3 were down-modulated, with at least 2-fold difference, when the expression of Vav1 was silenced with specific siRNAs in BT-474 cells (Figure 4A). The up-regulated genes include CTNNB1, GSC and TWIST1, encoding for transcription factors known to be activated during breast tumor metastasis, and AHNAK and AKT1, variously involved in tumorigenesis and cell motility (Table 3). On the other hand, the down-modulated genes codify for proteins involved in cell migration and invasion (SNAI2 and B2M) as well as for proteins (TFPI2) with a tumor suppressor role in breast cancer (Table 3). 8 genes, none of which in common with BT-474 cells, were up-regulated, with at least 2-fold difference, when the expression of Vav1 was silenced in MDA-MB-231 cells (Figure 4A). With exception of SNAI3, all the up-regulated genes are known to be involved in breast tumor malignancy and include the transcription factor FOXC2, PDGFRB and members of the WNT family, whose products are known to sustain the metastatic process of breast tumors (Table 3). In both cell lines, no significant effects of Vav1 down-modulation on the expression of genes codifying for the EMT markers Vimentin and E-cadherin were revealed (data not shown). Also the expression of ESR1 increased when Vav1 was down-modulated in both BT-474 cells (less than 2 fold, then not reported in Figure 4A) and MDA-MB-231 cells (Figure 4A), even though a further increase of the protein in the ER-positive BT-474 cells and its appearance in the ER-negative MDA-MB-231were not observed (Figure 4B).

**Table 3:** Roles in breast cancer of proteins codified by genes whose expression is regulated by Vav1.

Cell lines	Gene name	Effect of Vav1 siRNAs	Codified protein	Main function/Biological process	Known roles in breast cancer cells
**BT-474**	AHNAK AKT1 COL5A2 CTNBB1 GSC JAG1 KRT7 PLEK2 TWIST1 CALD1 SNAI2 TFPI2 VCAN B2M	UP UP UP UP UP UP UP UP UP DOWN DOWN DOWN DOWN DOWN	AHNAK nucleoprotein Akt Collagen, type V, α 2 β-catenin Goosecoid homeobox Jagged1 Keratin 7 (CK7) Pleckstrin 2 Twist homolog 1 Caldesmon 1 Snail homolog 2(SLUG) Tissue factor pathway inhibitor 2 Versican β-2-microglobulin	Scaffold protein/Cell motility Protein kinase/Cell growth and differentiation ECM component/ECM remodelling, Cell adhesion CAM/Cell growth and differentiation, Cell adhesion Notch ligand/Differentiation and development Notch ligand/Cell growth and differentiation (Angiogenesis) Binding protein/Cell motility Binding protein/Cell motility Transcription factor/Differentiation and development Binding protein/Cell motility Transcription factor/Differentiation and development ECM component/ECM remodelling ECM component/Cell growth and differentiation Binding protein/Cell growth and differentiation	Cell migration and invasion (30) Tumorigenesis (31) Tumor progression (32) Metastasis (33) Metastasis (34) Tumor marker (35) Tumor marker (36) Unknown Metastasis (37) Unknown Cell migration and invasion (38) Tumor suppressor (39) Metastasis (40) Cell migration and invasion (41)
**MDA-MB-231**	ESR1 FOXC2 KRT14 MMP3 PDGFBR SNAI3 WNT11 WNT5B	UP UP UP UP UP UP UP UP	Estrogen Receptor 1(ERα) Forkhead box C2(FOXC2) Keratin 14 (CK14) Matrix metallopeptidase 3 PDGF β-receptor Snail homolog 3 Wnt11 Wnt5B	Transcription factor/ER signalling Transcription factor/ECM remodelling Binding protein/Differentiation and development Proteolytic enzyme/ECM remodelling Membrane receptor/Differentiation and development Transcription factor/Differentiation and development Frizzled receptor‘s ligand/Differentiation and development Frizzled receptor‘s ligand/Differentiation and development	Tumorigenesis (25) Metastasis (42) Tumor marker (36) Tumor marker (43) Metastasis (44) Unknown Cell migration (45) Metastasis (45)

**Figure 5:**
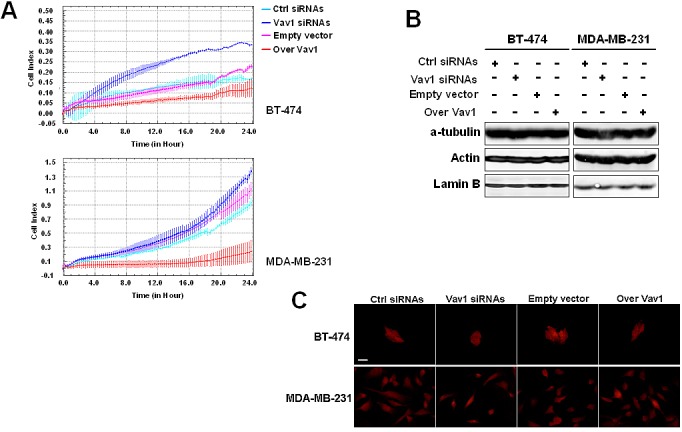
Involvement of Vav1 in *in vitro* malignant features of breast tumor cells (A) Dynamic monitoring of invasion through Matrigel using the xCELLigence system for the indicated time on BT-474 and MDA-MB-231 cells transfected with siRNAs specific for Vav1 (Vav1 siRNAs) or with a construct expressing human Vav1 (over Vav1). Error bars indicate ± SD. The data are representative of three experiments. Cells under the same experimental conditions were subjected to Western Blot analysis of cytoskeleton components (B) and to F-actin staining by using TRITC-coupled phalloidin (C). Lamin B was used as internal control of loaded proteins. The data are representative of three experiments. Bar = 20 μm.

The actual role of Vav1 in modulating malignant properties of breast tumor-derived cells was investigated in both BT-474 and MDA-MB-231 cells that were transiently transfected with siRNAs specific for Vav1 (Supplementary Figure 1A) or with a construct expressing the entire human Vav1 protein (Supplementary Figure 1B) and subjected to analysis of their invasion kinetics. As reported in Figure 5A, the silencing of Vav1 expression induced a slight but significant invasion of the poorly invasive BT-474 cells and significantly increased the invasion capability of MDA-MB-231 cells. On the other hand, the over-expression of Vav1 strongly counteracted the invasion ability of MDA-MB-231 (Figure 5A). To establish if Vav1 may modulates invasive properties of breast tumor-derived cells by affecting cytoskeleton, the expression of α-tubulin and actin (Figure 5B) as well as actin polymerization (Figure 5C) were investigated, failing to show significant effects due to levels of Vav1.

**Figure 6:**
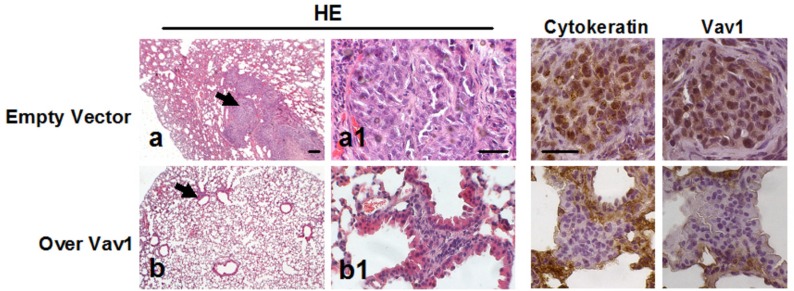
Role of Vav1 in preventing the development of secondary tumors *in vivo* Representative images of lung tissue sections from mice following intravenous injection with MDA-MB-231 cells transfected with an empty vector or with a construct expressing human Vav1 (Over Vav1). Sections were stained with hematoxylin/eosin (HE) or subjected to immunohistochemical analysis with anti-cytokeratin (Cytokeratin) and anti-Vav1 (Vav1) antibodies. Arrows in a and b indicate cell aggregates, shown at higher magnification in a1 and b1, respectively. Bar = 50 μm.

The possible role of Vav1 in modulating the potential of MDA-MB-231 cells to form secondary tumors was investigated *in vivo* by employing the tail vein injection assay, which allowed to assess the ability of MDA-MB-231 cells over-expressing Vav1 to produce lung tumors in mice. After 12 weeks from the injection no detectable macrometastases were observed on the surface of lungs of animals receiving cells transfected with the empty vector or cells over-expressing Vav1 (data not shown). However, the analysis of hematoxylin and eosin (HE)-stained sections revealed that 5/7 mice receiving empty vector transfected cells developed lung macrometastases or discrete nodules. The human origin of these tumor masses was confirmed by the immunohistochemical analysis of the human Cytokeratin and Vav1 (Figure 6). On the contrary, none of the mice receiving MDA-MB-231 cells over-expressing Vav1 developed visible cell aggregates of human origin in the lungs (Figure 6), indicating that high levels of Vav1 counteracted the metastatic efficiency of MDA-MB-231 cells.

## DISCUSSION

As for other signalling proteins normally restricted to hematopoietic cells, the ectopic expression of Vav1 is associated with a tumoral phenotype, suggestive for a role of this protein in solid tumors. Vav1 was found to be expressed in the majority of human neuroblastomas [7] and pancreatic ductal adenocarcinomas [8], in over 40% of human primary lung cancers [11] and melanomas [10] and in greater than 50% of ovarian cancers [12]. Concerning breast cancer, *Vav1* was reported to be one of the genes with altered expression in medullary breast cancers [9] and a *Vav1* transcript higher than in normal tissue was reported in different breast cancer subtypes [13]. In a recent study, 62% of primary breast tumors of various grades and stages were found to express Vav1 protein [14]. Despite the presence of transcripts for Vav1 in a large number of breast tumor-derived cell lines, the same study failed to detect significant amounts of the protein in lysates from the same cells [14].

Here we have reported an immunohistochemical analysis of Vav1 expression in 137 primary node negative early breast cancers representing all the most frequent breast tumor histotypes. We found that, even if to a variable extent, almost all the tumors resulted positive for Vav1 and, as in hematopoietic cells [4, 17], the protein showed a localization also inside the nuclear compartment. The subcellular localization of Vav1 was also investigated in 5 human breast tumor-derived cell lines (BT-474, MCF7, MDA-MB-453, MDA-MB-468 and MDA-MB-231) representing the most frequent subtypes of breast cancer in terms of immunoprofile, molecular phenotype and *in vivo* tumorigenicity [18, 19]. The analysis of purified nuclei allowed to confirm the peculiar accumulation of Vav1 inside the nucleus of breast tumor cells, an occurrence that may justify the little amount of the protein found in total lysates from the same cell lines [14].

Due to the main nuclear localization of Vav1, the relationship between nuclear Vav1 and malignant features of breast tumors was then investigated; however, we failed to identify significant correlations with the most used clinic-pathological parameters, including tumor grade and receptors status. This in contrast with the whole cellular level of Vav1 that, in a study less extensive and including a great variety of tumor stages, was reported to be positively correlated with the expression of ER and PR [14].

Despite the absence of any correlation between nuclear Vav1 and positivity/negativity to ER, we have found that the ER status of primary tumors seems to be at the basis of a close relationship of Vav1 with menopausal status of breast tumor patients. In fact, only in ER+ tumors, high amounts of Vav1 inside the nucleus of tumor cells correlate with the post-menopausal status. This was also true when the cut-off value for ER positivity was reduced to nuclear staining as low as 1% of cells, as suggested by the most recent guidelines [15]. Since low levels of nuclear Vav1 are present in tumors from pre/peri-menopausal patients, the nuclear amounts of Vav1 seem to inversely correlate with the estrogens availability. This suggests that, inside the nucleus of breast tumor cells, Vav1 may compete with the classical nuclear activity of ERα, a ligand-dependent transcription factor known to play a crucial role in breast cancer development and/or progression [20]. These data also suggest that in breast cancers Vav1 plays a role distinct from Vav3, which is known to complex with ERα and thus to enhance ERα activity [21].

The amount of Vav1 inside the nucleus of breast tumor cells seems to be independent also from the diversity of the different clinicopathological subtypes, identified by the criteria of Goldhirsch et al. [16] and currently of practical use for therapeutic purposes. The absence of a correlation between nuclear Vav1 and the different molecular subtypes was confirmed in breast tumor-derived cells, since Luminal B (BT-474), HER2 (MDA-MB-453) and Claudin-low (MDA-MB-231) cells show similar levels of the protein, indicating that the de-regulation of Vav1 expression and the accumulation of the protein inside the nucleus of breast tumor cells may not be uniquely correlated to any of the known markers of tumorigenic/metastatic properties of breast tumors [16].

Concerning the prognostic value of the ectopic expression of Vav1 in solid tumors, it was reported to be inversely correlated with positive prognosis of PDA patients [8] and associated with decreased survival rates in early-stage patients affected by epithelial ovarian cancer [12]. In breast tumors, contrarily to Vav2 and Vav3, that play synergistic roles in breast cancer by sustaining tumor growth, neo-angiogenesis and metastasis [22], the *Vav1* transcript seems to be higher in tumors from patients that remained disease free than in patients who developed recurrence [13]. Here, we demonstrate that high amounts of Vav1 protein inside the nuclear compartment of cells from primary early breast tumors are positively correlated with the absence of recurrence, regardless of the histological subtypes of primary tumors. In particular, nVav1^high^ tumors were associated with a significantly lower incidence of distant relapse and nuclear expression of Vav1 resulted to be an independent prognostic factor influencing DRFS in the whole examined tumors collection and regardless the different molecular subtypes. This is particularly interesting if we consider that not always the clinical management of breast cancer can be supported by the individual molecular typing and that the practically used cliclinicopathological classification [16] must deal with distinct cases within the various subtypes.

At variance with other solid tumors, in which the malignancy-associated role of Vav1 has been variously linked to its GEF activity [8, 11, 13], opposite effects for Vav1 on Rac1 activation were reported in AU565 and MCF7 breast tumor-derived cell lines, also in the presence of cytoskeleton changes [14]. On the other hand, opposite effects on apoptosis were observed in the two cell lines, possibly related to p53 availability [14], suggestive of diverse signalling pathways involving Vav1 in breast tumor cells, also independent of its cytoplasm-confined GEF activity.

Roles of Vav1 alternative to the GEF activity and involving the nuclear compartment were studied exclusively in cells from myeloid leukemia, in which the protein was implicated in the transcriptional machinery by direct interaction with, or as a facilitator of, transcription factors [4, 6]. In cells derived from Acute Promyelocytic Leukemia (APL), Vav1 is part of interconnected networks of functionally related proteins ended to regulate different aspects of gene expression. In particular, a direct interaction of nuclear Vav1 with transcription factors was demonstrated, as well as its role in regulating the formation of DNA-associated complexes [23]. Consistent with a role in modulating the transcriptional machinery, Vav1 carries inside the nuclear compartment of APL-derived cells molecules involved in modulation of mRNA production and stability [24].

The high levels of Vav1 we have found inside the nucleus and its accumulation in sub-nuclear structures in which it co-localizes with the speckle marker SC-35 [25] clearly suggested a role for Vav1 in modulating nucleic acid metabolism also in breast tumor cells. The role of Vav1 in modulating the expression of a focused panel of genes involved in tumor progression was investigated in the non-metastatic BT-474 and in the moderately metastatic MDA-MB-231 cells, both showing high amounts of Vav1 at nuclear level. Two different groups of genes were found to be modulated in the two cell lines as a consequence of the down-regulation of Vav1. The majority of up-regulated genes in BT-474 cells and almost all of the genes up-regulated in MDA-MB-231 cells are known to sustain progression of breast cancer, while down-regulated genes in BT-474 cells are known to have a tumor suppressor role [20, 26-41]. These data clearly indicate that the presence of Vav1 inside the nucleus of breast tumor-derived cells, and likely in mRNA processing structures, may have role in modulating the expression of malignancy-related genes. It might be thus speculated that high levels of nuclear Vav1 in primary breast tumors, regardless the phenotype and the molecular subtype, may counteract tumor progression by negatively modulate the expression of genes involved in tumor invasion and/or metastasis.

The actual role of Vav1 in modulating invasive properties of breast tumor-derived cells was demonstrated by performing Real-time cell invasion assays with both BT-474 and MDA-MB-231 cell lines. Our results demonstrated that the silencing of Vav1 expression induced invasion of the non-invasive BT-474 and significantly increased the invasion capability of the highly invasive MDA-MB-231. On the other hand, when the amount of Vav1 was further increased by over-expression of a full length human protein, the *in vitro* invasion capability of MDA-MB-231 cells was drastically reduced. These events are not a consequence of the known capability of Vav1 to affect cytoskeleton components [3, 42, 43] but seem mainly related to its ability to modulate the expression of malignancy-related genes.

Over-expression of Vav1 in MDA-MB-231 cells strongly reduced their potential to form secondary tumors *in vivo,* as demonstrated by the tail vein injection assay. This approach, which allows to assess the ability of cells to produce lung tumors in immunodeficient mice without performing intravasation [44], showed that almost all mice receiving empty vector transfected cells and none of the mice receiving MDA-MB-231 cells stably over-expressing Vav1 developed lung macro-metastasis or discrete nodules, indicating that high levels of Vav1 counteract the metastatic efficiency of this cell line.

It is well known that metastasis is a multistep process by which primary tumor cells invade adjacent tissue, enter the blood stream, extravasate into the surrounding tissue parenchyma and finally form secondary lesions. The precise mechanisms involved in the transition of non-invasive tumour cells into cells with metastatic potential are still largely unknown and controversial is the requirement of an epithelial to mesenchymal transition (EMT)-like process for metastasis of solid tumors [45]. At any rate, the hallmark of EMT in cancer seems to be the down-regulation of E-cadherin, which is also thought to be a repressor of invasion and metastasis [46]. In addition, a group of transcription factors have been demonstrated to be capable of orchestrating EMT programmes in cancer progression, including direct transcriptional repressors of E-cadherin, like Snail (SNAI1), Slug (SNAI2), ZEB2 (SIP1) and others transcription factors like Twist, ZEB1 and FOXC2, whose role in metastasis depends on the microenvironment, cell type and other signalling within the cells [34].

A role for Vav1 in modulating EMT has been reported in cells derived from ovarian cancer in which, at variance with breast tumor, the expression of the protein correlates with a poor prognosis of early stage patients [12]. In particular, overexpression of Vav1 in ovarian tumor-derived cells induces down-regulation of E-cadherin and up-regulates Snail and Slug, suggesting the use of this protein as a potential therapeutic target to prevent metastasis of ovarian cancer [12]. In breast tumor-derived cells, we failed to observe any significant correlation between Vav1 and the expression levels of the epithelial marker E-cadherin as well as of the mesenchymal protein Vimentin. In fact, high levels of Vav1, particularly nuclear, are shown by the epithelial-like pre-EMT BT-474 cell line, wich also express high levels of E-cadherin, and by the spindle-like post-EMT MDA-MB-231 cells, that are E-cadherin negative and express high levels of Vimentin [18]. In addition, when we down-modulated the expression of Vav1 in both cell lines, we could not find any difference in the expression levels of genes codifying for E-cadherin and Vimentin. On the other hand, except for Twist, that in breast tumors has been reported to promote metastasis by regulating the formation of invadopodia [33], no significant differences in expression levels of transcription factors directly involved in the regulation of E-cadherin were found in both cells lines as a consequence of Vav1 silencing. Although loss of E-cadherin in human breast cancer may not be causal for EMT and even not a necessity [47], these data indicate that the effects of Vav1 on metastatic potential of breast tumor cells are not mediated by this junction protein. On the other hand a role for Vav1 in regulating EMT in breast cancer cannot be excluded, as suggested by the up-regulation of genes whose expression sustains EMT [34] as a consequence of silencing of Vav1. In addition, even if the occurrence and significance of EMT in breast cancer is still under debate, a recent work suggests that, also in this solid tumor, the formation of clinically detectable metastases requires a process opposite to the initial EMT at the primary tumour site, consisting in a mesenchymal to epithelial transition (MET) at a distant metastatic site [48]. Since the ability of MDA-MB-231 cells, that have already performed the EMT process [18, 19], to form secondary tumors in mice is strongly inhibited by over-expression of Vav1, a specific role of this protein may be related to the efficiency of tumor cells to perform the last part of the metastatic process, consistent in colonization of parenchyma of distant organs.

In conclusion, our findings provide new insights into the role of Vav1 in solid tumors and indicate an unprecedented function for this protein in reducing the ability of breast tumor cells to form metastasis. Also unique is the role as an independent positive prognostic factor played by Vav1 in early-stage breast tumors, regardless cancer subtypes. If we consider that a breast cancer patient has very good chances of a disease-free survival if the cancer is treated early, the monitoring of Vav1 in early breast tumors may be beneficial in selecting the more appropriate therapy.

## MATERIALS AND METHODS

### Patients and tumors

The current study included 137 primary infiltrating breast cancers from N0 patients with T1/T2 tumors, diagnosed between 1994 and 2001 at the Regina Elena National Cancer Institute, Rome, Italy, and presenting primary unilateral breast carcinoma. The study was reviewed and approved by the ethics committee of the “Regina Elena” National Cancer Institute and written informed consent was obtained from all patients. Patient and tumor characteristics are summarized in Table 1.

All patients received radiation therapy. Forty patients received hormonal therapy and 128 patients were treated with adjuvant chemotherapy (followed or not by hormonal therapy). Patients with HER-2-positive tumors did not receive trastuzumab, because it was not used in breast tumor therapy in the study period.

Follow-up data were available for all patients and were obtained from institutional records or by the referring physicians. The median follow-up was of 94 months (range 19-171 months). During follow-up, 8 patients (5.8%) developed a local recurrence and distant metastases were observed in 16 cases (11.7%).

Tumors were evaluated for their receptors status and proliferation levels as previously reported [49].

As all of our cases were diagnosed prior to 2010, cut-offs for estrogen receptors (ER) and progesterone (PR) receptors were 10% and samples were classified as positive if ≥10% of positive staining cells were counted. A specimen was considered with high levels of Ki-67 expression if ≥14% of tumor nuclei stained positively. HER-2 membranous staining was scored according to Herceptest (Dako, Glostrup, Dk) and classified as positive if the intensity was scored 3+, with more than 30% of cells showing complete membrane staining, or if the intensity was scored 2+ in presence of an amplification of the HER-2 gene as assessed by fluorescent in situ hybridization.

### Immunohistochemical analysis of Vav1

All reagents were from Sigma (St Louis, MO) unless otherwise indicated. Tissue microarrays (TMAs) were constructed as described by Lattanzio et al. [49]. For analysis of Vav1 expression, formalin-fixed, paraffin embedded arrays were deparaffinized by means of xylene and rehydrated in distilled water through graded alcohols (ethanol 100, 95 and 70%, respectively). Antigen retrival was performed in citrate buffer pH6 and heat (98°C) for 20 min. Arrays were then subjected to immunohistochemical analysis with an anti-Vav1 antibody using the UltraVision LPValue Detection System: HRP Polymer (Ready-To-Use) (Thermo Fisher Scientific, Waltham, MA), according to the manufacturer's instruction.

In particular, the slides were incubated in 3% (v/v) H_2_O_2_ to block endogenous peroxidases and the Ultra V Block reagent (Thermo Fisher Scientific) was used to reduce background. Afterwards, the arrays were incubated at room temperature for 90 min with anti-Vav1 rabbit polyclonal IgG (1:100) (Santa Cruz Biotechnology, Santa Cruz, CA) in Large Volume UltrAbDiluent (Thermo Fisher Scientific). Localization was detected by the addition of substrate/chromogen mix (DAB Quanto, Thermo Fisher Scientific) and the tissue microarrays sections were finally counterstained with Mayer's hemallume solution (Bio-Optica, Milan, I) and rinsed for approximately 10 min in tap water. Negative controls were obtained by omitting the primary antibody. Each tissue sample, after hybridization with the anti-Vav1 antibody, was analyzed by an optical microscope (Carl Zeiss Axiophot 100, Zeiss, Göttingen, D) equipped with a Nikon Digital Sigh DS Vi1 camera (Nikon, Lijnden, NL) and the stain intensity was estimated with ImageScope software (Aperio, Vista, CA) by analysis of acquired images. For all samples, three different areas, each containing approximately 100 cells, were analyzed and tumors were considered negative for Vav1 when less than 5% of cells were stained. Positive tumors were categorized on the basis of their nuclear amount of Vav1, arbitrarily defined as low or high expression. As a general criterion, a 60% cut-off was selected, corresponding to the 50th percentile, as previously reported [49], and a specimen was considered with high levels of nuclear Vav1 expression if ≥ 60% of nuclei were highly expressing.

### Breast tumor-derived cell lines

The breast cancer-derived cell lines MDA-MB-231, MDA-MB-453, MDA-MB-468 and MCF7 cells were purchased from the American Type Culture Collection (Rockville, MD). The BT-474 cell line was from ICLC (Genova, I). MDA-MB-231, MDA-MB-453, MDA-MB-468 and MCF7 cell lines were grown in Dulbecco's modified Eagle's medium (DMEM, Gibco Laboratories, Grand Island, NY) supplemented with 10% fetal bovine serum (FBS, Gibco Laboratories). BT-474 cells were maintained in RPMI 1640 growth medium (Gibco Laboratories) supplemented with 10% FBS, 1 mM Na pyruvate and 0.01 mg/ml bovine insulin. Cells were grown at 37 °C in a humidified atmosphere of 5% CO_2_ in air. Subconfluent cells were counted daily and cell morphology was evaluated using an inverted phase-contrast microscope (Nikon).

Cells were monthly tested for mycoplasm and other contaminations and quarterly subjected to cell identification by single-nucleotide polymorphism.

### Isolation of membrane-deprived nuclei and immunochemical analysis

Purification of nuclei deprived of nuclear membrane was performed essentially as previously reported [50]. Cells were resuspended in 10 mMTris–HCl, pH 7.4, 2 mM MgCl_2_, 10 mM NaCl, protease (0.5 mM PMSF, 1 μg/mL Aprotinin, 1 μg/mL Leupeptin) and phosphatase (1mM Na_3_VO_4_) inhibitors, all from Calbiochem (La Jolla, CA), incubated 10 min at room temperature and 10 min in water/ice mix and then passed 4 times through a syringe with a 25-gauge needle. The nuclear pellet was washed in 10 mM Tris–HCl, pH 7.4, 5 mM MgCl_2_, 10 mM NaCl added of protease and phosphatase inhibitors, suspended in Laemmli's SDS sample buffer and then subjected to Western blot analysis. The absence of the outer nuclear membrane as well as of cytoplasmic contaminations was assessed by ultrastructural analysis and marker enzyme assays, as previously described [50].

For Western blot analysis, total lysates (50 μg protein) from cells and membrane-depleted nuclei were separated on 7.5% polyacrylamide denaturing gels and blotted to nitrocellulose membranes (GE Healthcare Life Science, Little Chalfont, UK). The membranes were incubated with specific antibodies directed against Vav1, Lamin B1 and ERα (Santa Cruz Biotechnology) and against α-tubulin, β-tubulin and actin (Sigma), as previously reported [42]. All membranes were then incubated with peroxidase-conjugated secondary antibodies and revealed by using the ECL system (PerkinElmer, Boston, MA), according to the manufacturer's instructions. The chemiluminescence bands were collected with ImageQuant™ LAS 4000 biomolecular imager (GE Healthcare) and the densitometric analysis was performed by means of Image Quant TL software (GE Healthcare).

### Immunocytochemical analysis

The different breast tumor-derived cell lines, grown onto glass slides, were fixed with freshly prepared 4% paraformaldehyde (10 min at room temperature), washed in PBS (5 min) and reacted with the anti-Vav1 and/or anti-SC-35 (Sigma) antibodies in NET gel for 3 h at room temperature, following a previously reported procedure [51].

To analyze F-actin, fixed cells were washed with PBS, permeabilized with 1% Triton X100 in PBS for 5 min at room temperature, incubated with 1% BSA for 30 min and then stained with TRITC-conjugated phalloidin, in PBS for 30 min at room temperature in the dark.

After two washes with NET gel or PBS, samples were incubated 1 min with 0.5 μg/ml 4',6-diamidino-2-phenylindole (DAPI), then washed in PBS, dried with ethanol, mounted in glycerol containing 1,4-diazabicyclo [2.2.2] octane (DABCO) to retard fading.

Fluorescent samples were analyzed with a Nikon Eclipse TE2000-E microscope (Nikon, Melville, NY), acquiring cell images by the ACT-1 software for a DXM1200F digital camera (Nikon), or with a Zeiss 510 laser-scanning confocal microscope (Carl Zeiss, Oberkochen, D). Confocal images were processed by using the LSM Image Browser (Zeiss).

### Modulation of Vav1 expression

Three small interfering RNA (siRNA) sequences targeting the mRNA for Vav1 were synthesized by Santa Cruz Biotechnology. Exponentially growing BT-474, MDA-MB-453 and MDA-MB-231 cells were transfected with a mixture of Vav1 siRNAs and with non-silencing control siRNAs (Santa Cruz Biotechnology), as previously reported [52]. As a control of transfection efficiency, which resulted always higher than 70%, a non-silencing fluorescein-labeled duplex RNA (QIAGEN S.p.A., Milan, I) was used. For Vav1 over-expression, the above reported cells were transfected with a pEF plasmid expressing the human full-length Vav1 following a previously reported procedure [52]. The transfected cells were incubated at 37°C in a 5% CO_2_ atmosphere for 24-48 h and then subjected to immunochemical and immunocytochemical analysis.

To obtain MDA-MB-231 cells stably expressing Vav1, after 48 h from transfection, cells were cultured for 3 weeks adding 1mg/ml G418 to the growth medium. The resistant cells were maintained in culture with a medium containing 0.1 mg/ml G418 and then used for *in vivo* experiments.

### Real-time cell invasion assays

Cell invasion was evaluated by means of the xCELLigence RTCA System (Real-Time Cell Analyzer System, Roche Applied Science, Mannheim, D), developed to monitor cell events in real time by measuring the electrical impedance produced by cells, essentially as previously reported [52]. In particular, 40.000 cells / well were seeded onto the top chambers of CIM-16 plates (Roche) covered with a layer of Matrigel (BD Biosciences, San Josè, CA) diluted 1:20. The bottom chambers were filled with medium containing 10% serum and the signal detection was programmed every 15 min for a total of 24 h. Impedance values were expressed as a dimensionless parameter (cell index, CI).

### PCR Array analysis

High-quality total RNA from BT-474 and MDA-MB-231 cells transfected with control siRNAs and with siRNAs specific for Vav1 was extracted with RNeasy^®^ Micro Kit (Qiagen) and was utilized (500 ng) for single stranded cDNA synthesis using RT^2^ First Strand Kit (Qiagen), as reported by the manufacturer. The cDNAs were employed as templates for SYBR^®^ Green based RT^2^-qPCR Epithelial-Mesenchymal Transition (SABiosciences Corp., Frederick, MD) to profile the expression of 84 genes related to signallig activated during metastasis (listed in Supplementary Table 1), in accordance with manufacturer's instructions. Termal cycling and fluorescence detection were performed using a Bio-Rad CFX96™ sequence detection system (Bio-Rad Laboratories, Hercules, CA) and the data were analyzed by using a SABiosciences software (SABiosciences).

### Metastatic animal model

All procedures for animal experiments were approved by the Committee on the Use and Care of Animals and performed in accordance with the institution guidelines (CEISA, Chieti, I).

To produce experimental metastasis, 6 week old female BALB/c nude mice (Charles River Laboratories Italia, Lecco, I) were injected intravenously with MDA-MB-231 cells over-expressing human Vav1 or the empty vector (2 × 10^6^ in 0.1 ml PBS) via tail veins (7 mice were set for each group). Throughout the experiments, mice were maintained with free access to pellet food and water.

After 12 weeks, the mice were euthanized and the lungs were removed and fixed with 4% paraformaldehyde. Paraffin embedded sections were stained with HE and the number of discrete tumors as well as the presence of visible nodules were evaluated.

Paraffin embedded sections of lung from mice receiving cells either transfected with empty vector or expressing human Vav1 were subjected to immunohistochemical analysis of human cytokeratin and Vav1 using the UltraVision LPValue Detection System, as above reported.

### Statistical analysis

The relationships between Vav1 expression and clinicopathological parameters were assessed by Pearson's χ2 or Fisher's exact test, as appropriate. DFS was defined as the time from surgery to the first of the following events: tumor recurrence at local site or at distant sites. Local (LRFS) and distant relapse-free survivals (DRFS) were the times from surgery to the occurrence of relapse at local and distant sites, respectively. Kaplan-Meier plots were used to show the survival in specified cohorts and the log-rank test to check for equality of survival curves.

The association of Vav1 expression with outcome, adjusted for other prognostic factors, was tested by Cox's proportional hazards model. The following covariates were included in the multivariate DFS models: tumor size, tumor grade, ER, PR, Ki-67, HER-2 and Vav1 status. Statistical analysis was performed by SPSS Version 15.0 (SPSS, Chicago, IL) and *P* ≤ 0.05 was considered statistically significant.

## SUPPLEMENTARY FIGURE AND TABLE


