# Supplementary material for: High nuclear level of Vav1 is a positive prognostic factor in early invasive breast tumors: a role in modulating genes related to the efficiency of metastatic process
Source: Oncotarget. 2014 May 25;5(12):4320–36. doi: 10.18632/oncotarget.2011 (PMC4147326; doi:10.18632/oncotarget.2011)
Supplement: Supplementary file 1 [file oncotarget-05-4320-s001.pdf]

High nuclear level of Vav1 is a positive prognostic factor in early invasive breast tumors: a role in modulating genes related to the efficiency of metastatic process – Grassilli et al

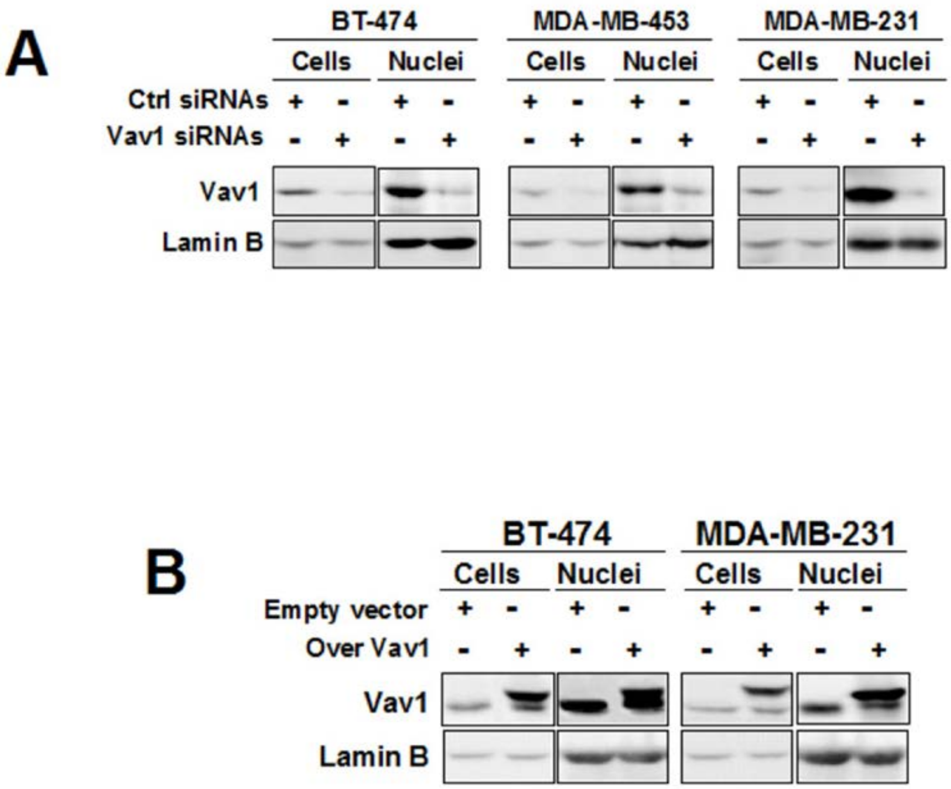

Supplementary Figure 1: Modulation of Vav1 expression in breast tumor cells. (A) Immunochemical evaluation of Vav1 expression on cellular and nuclear lysates from BT-474, MDA-MB-453 and MDA-MB-231 cells transfected with siRNAs specific for Vav1 (Vav1 siRNAs). A non-silencing scramble siRNAs was used as a control (ctrl siRNAs). Lamin B content constituted an internal control of protein loading. (B) Evaluation of Vav1 expression by Western Blot analysis of cellular and nuclear lysates from BT-474 and MDA-MB-231 cells transfected with an empty vector or with a construct expressing human Vav1 (over Vav1). Lamin B was blotted as a control for loaded proteins. The data are representative of three experiments.

Supplementary Table 1	
Gene table: Human EMT RT^2 Profiler PCR Array	
**Position**	**Symbol**
A01	AHNAK
A02	AKT1
A03	BMP1
A04	BMP7
A05	CALD1
A06	CAMK2N1
A07	CAV2
A08	CDH1
A09	CDH2
A10	COL1A2
A11	COL3A1
A12	COL5A2
B01	CTNNB1
B02	DSC2
B03	DSP
B04	EGFR
B05	ERBB3
B06	ESR1
B07	F11R
B08	FGFBP1
B09	FN1
B10	FOXC2
B11	FZD7
B12	GNG11
C01	GSC
C02	GSK3B
C03	IGFBP4
C04	IL1RN
C05	ILK
C06	ITGA5
C07	ITGAV
C08	ITGB1
C09	JAG1
C10	KRT14
C11	KRT19
C12	KRT7
D01	MAP1B
D02	MITF
D03	MMP2
D04	MMP3
D05	MMP9
D06	MSN
D07	MST1R

D08	NODAL
D09	NOTCH1
D10	NUDT13
D11	OCLN
D12	PDGFRB
E01	PLEK2
E02	PPPDE2
E03	PTK2
E04	PTP4A1
E05	RAC1
E06	RGS2
E07	SERPINE1
E08	SIP1
E09	SMAD2
E10	SNAI1
E11	SNAI2
E12	SNAI3
F01	SOX10
F02	SPARC
F03	SPP1
F04	STAT3
F05	STEAP1
F06	TCF3
F07	TCF4
F08	TFPI2
F09	TGFB1
F10	TGFB2
F11	TGFB3
F12	TIMP1
G01	TMEFF1
G02	TMEM132A
G03	TSPAN13
G04	TWIST1
G05	VCAN
G06	VIM
G07	VPS13A
G08	WNT11
G09	WNT5A
G10	WNT5B
G11	ZEB1
G12	ZEB2
H01	B2M
H02	HPRT1
H03	RPL13A
H04	GAPDH
H05	ACTB
H06	HGDC
H07	RTC

H08	RTC
H09	RTC
H10	PPC
H11	PPC
H12	PPC
